# Protective Effect of Hypercapnic Acidosis in Ischemia-Reperfusion Lung Injury Is Attributable to Upregulation of Heme Oxygenase-1

**DOI:** 10.1371/journal.pone.0074742

**Published:** 2013-09-10

**Authors:** Shu-Yu Wu, Min-Hui Li, Fu-Chang Ko, Geng-Chin Wu, Kun-Lun Huang, Shi-Jye Chu

**Affiliations:** 1 Institute of Aerospace and Undersea Medicine, National Defense Medical Center, Taipei, Taiwan; 2 Department of Internal Medicine, Taoyuan Armed Forces General Hospital, Taoyuan, Taiwan; 3 Department of Internal Medicine, Tri-Service General Hospital, National Defense Medical Center, Taipei, Taiwan; University of Rochester Medical Center, United States of America

## Abstract

Hypercapnic acidosis (HCA) has protective effects in animal models of acute lung injury, but the mechanism underlying the effect of HCA is unclear. Heme oxygenase-1 (HO-1) is an antioxidant enzyme that protects tissue from inflammation injury. We investigated whether HO-1 contributes to the protective effects of HCA in ischemia-reperfusion (IR)-induced lung injury. Typical acute lung injury in rats was successfully induced by 40 min of ischemia and 90 min of reperfusion in an isolated perfused lung model. The rat lungs were randomly assigned to the control group, IR group or IR + HCA group with or without zinc protoporphyrin IX (ZnPP), an HO-1 activity inhibitor. At the end of the experiment, bronchoalveolar lavage fluid (BALF) and lung tissues were collected to evaluate the degree of lung injury. In *in vitro* experiments, HO-1 siRNA transfected A549 cells were exposed to a normoxic or hypoxia-reoxygenation (H/R) environment in the presence or absence of HCA. IR caused significant increases in the pulmonary arterial pressure, lung weight to body weight and wet/dry ratios, lung weight gain, capillary filtration coefficient, lung injury scores, neutrophil infiltration, and concentrations of protein and TNF-α in the BALF. IR also induced degradation of inhibitor of nuclear factor (NF)-κB-α, increased IκB kinase (IKK)-β phosphorylation and nuclear translocation of NF-κB, and up-regulated HO-1 expression and activity. Furthermore, IR decreased Bcl-2 protein expression and increased the number of active caspase-3 stained cells. HCA treatment enhanced HO-1 expression and activity, and accordingly reduced IKK-NF-κB signaling, inhibited apoptosis, and significantly attenuated IR-induced changes. Treatment with ZnPP partially blocked the protective effect of HCA. In addition, HO-1 siRNA significantly reversed HCA-mediated inhibition of NF-κB signaling in A549 cells subjected to H/R. In conclusion, the protective effect of HCA in IR lung injury in rats was mediated in part by the anti-inflammatory and anti-apoptotic action of HO-1.

## Introduction

Hypercapnic acidosis (HCA) occurs when the pH drops below the normal range due to an excess of CO_2_ in the blood [1]. HCA can be attained purposely by the addition of a small fraction of inspired CO_2_ (typically 5-15%) or by increasing the dead space during mechanical ventilation. Low tidal volume ventilation improves the outcome of acute lung injury and acute respiratory distress syndrome and often leads to HCA [2]. Further analysis has revealed that HCA reduces 28-day mortality in patients receiving traditional ventilation [3]. HCA has proven protective in a variety of experimental lung injuries induced by free radical, ischemia-reperfusion (IR), mechanical ventilation, endotoxin and cecal ligation [4]. The underlying mechanisms of HCA benefits are complex, but attenuating the inflammatory reaction, suppressing nuclear factor -κB (NF-κB) activation, and inhibiting cell apoptosis play a pivotal role [1]. One recent study demonstrated that HCA had the ability to inhibit IκB-α degradation and NF-κB activation in ventilation-induced lung injury [5].

Heme oxygenase-1 (HO-1) catalyzes the rate-limiting step in oxidative degradation of heme to biliverdin, releasing iron and carbon monoxide (CO). This antioxidant enzyme is highly induced by oxidative stress including its substrate heme, heavy metals, cytokines, endotoxins, heat shock, and hypoxic ischemic injury; HO-1 plays a vital role in maintaining oxidative/antioxidant homeostasis [6]. Induction of HO-1 exerts significant protection in a number of preclinical animal models of disease and injury [6]. Furthermore, HO-1 protects against pulmonary IR lung injury. Reduced HO-1 protein expression in homozygous *ho-1* knockout mice is associated with severe lung injury [7]. Although HCA has antioxidant and anti-inflammatory actions, whether it induces HO-1 expression in lung tissues has not been investigated. Therefore, we designed this study to test the hypothesis that HCA induced HO-1 expression in lung tissue and conferred protection against IR-induced lung injury.

## Methods

### Isolated perfused lung preparation

The animals used for this study were cared for in accordance with the guidelines of the National Institutes of Health (National Academy Press, 1996), and approval for our study protocol was obtained from the National Science Council and Animal Review Committee of the National Defense Medical Center. Isolated rat lung was prepared as described previously [8]. In brief, male Sprague Dawley rats weighing 350 ± 20 g were anesthetized with intraperitoneal sodium pentobarbital (50 mg/kg). After insertion of a tracheal cannula, the lungs were ventilated with humidified gas containing 5% CO_2_ in air at a frequency of 60 cycles/min, a tidal volume of 3 ml, and an end-expiratory pressure of 1 cm H_2_O. After a median sternotomy was performed, heparin (1 U/g of body weight) was injected into the right ventricle and the pulmonary artery was cannulated and perfused with physiological salt solution containing 119 mM NaCl, 4.7 mM KCl, 1.17 mM MgSO_4_, 22.6 mM NaHCO_3_, 1.18 mM KH_2_PO_4_, 1.6 mM CaCl_2_, 5.5 mM glucose, and 50 mM sucrose. A wide-bore cannula was placed in the left atrium through the left ventricle to collect the effluent perfusate for recirculation. The perfusion rate was kept at 8–10 ml/min by a roller pump. The preparation was placed on an electronic balance with the isolated lungs remaining *in situ*. Both the pulmonary arterial pressure (PAP) and the left atrial pressure, which represents the pulmonary venous pressure (PVP), were monitored from side arms of the inflow and outflow cannulae. The pH, PCO_2_, and PO_2_ levels in the perfusate were measured from the side hole in the left atrial cannula, using an ABL 800FLEX blood gas analyzer (Radiometer, Copenhagen, Denmark).

### Microvascular permeability

An index of microvascular permeability to water (*K*_f_) was determined from the lung weight change induced by the elevation of venous pressure. During ventilation and lung perfusion, the PVP was rapidly elevated by 10 cmH _2_O for at least 7 min. The slow, steady phase of weight gain as a function of time (ΔW/ΔT) was plotted on semilogarithmic paper. The slow component then was extrapolated to zero time to obtain the initial rate of transcapillary filtration. From this plot, K_f_ was defined as the y-intercept (in g ·min^-1^) divided by the PVP (10 cmH _2_O) and lung weight, and it was expressed in whole units of g·min^-1^·cmH _2_O^-1^ × 100 g [9].

### Measurement of lung weight / body weight and wet / dry weight ratios

After the experiments, the right lung was removed from the hilar region and the wet weight was determined for calculation of the lung weight / body weight (LW/BW) ratio. A part of the right upper lung lobe was placed in an oven at 60°C for 48 h to allow for determination of the wet / dry (W/D) weight ratio.

### Protein concentration and tumor necrosis factor -α in bronchoalveolar lavage fluid

Bronchoalveolar lavage fluid (BALF) was obtained at the end of the experiment by irrigating the left lung twice with 2.5 ml of saline. The fluid was centrifuged at 200 × *g* for 10 min, and the concentration of protein in the supernatant was determined using the bicinchoninic acid (BCA) protein assay (Pierce, Rockford, IL, USA). Tumor necrosis factor -α (TNF-α) in the BALF was determined using a commercially available ELISA kit (R&D Systems Inc., Minneapolis, MN, USA).

### Western blot analysis

Cytoplasmic and nuclear proteins were extracted from frozen lung tissue with the Nuclear/Cytosol Extraction kit (BioVision, Inc., Mountain View, CA, USA) according to the manufacturer’s instructions. Protein concentrations were determined using the BCA protein assay (Pierce). Equal amounts of lung homogenates (30 µg/lane) were fractionated on 10%-12% sodium dodecyl sulfate polyacrylamide gel electrophoresis gels, and transferred to Hybond polyvinylidene fluoride membranes. The membranes were blocked by incubation in phosphate-buffered saline (PBS) containing 0.1% Tween 20 (PBST) and 5% nonfat milk for 1 h at room temperature. Blots were incubated with rabbit anti-HO-1 (1:5000, Enzo Life Sciences Inc., San Diego, CA, USA), Bcl-2, anti-NF-κB p65, anti-IκB-α and anti-phosphorylated IκB kinase (IKK)-α/β polyclonal antibodies and anti-IKKβ polyclonal antibodies (diluted 1:1000; Cell Signaling Technology, Danvers, MA, USA) overnight at 4 °C. The blots were then washed in PBST three times for 10 minutes. Blots were incubated with horseradish peroxidase-linked anti-rabbit IgG (1:40000) or anti-mouse IgG (1:50000) for 1 h at room temperature, then washed three times in PBST for 10 min. Bands were visualized using enhanced chemiluminescence reagents and by exposing the blot to X-ray film. The blots were then stripped and incubated with an anti-TATA antibody (for nuclear protein, diluted 1:1000; Abcam, Cambridge, MA, USA) or anti-β-actin antibody (for cytoplasmic protein, diluted 1:10000; Sigma, St. Louis, MO, USA) to ensure equal loading. The ratios of the band intensities were calculated.

### Activated caspase-3 immunohistochemistry

Formalin-fixed paraffin sections (4-µm) were deparaffinized before antigen retrieval and endogenous peroxidase was blocked using 3% H_2_O_2_ in methanol for 15 min. The slides were then incubated for 60 min with a polyclonal antibody (1 : 200 dilution; Cell Signaling Technology) that specifically recognizes the large fragment (17/19 kD) of activated, but not full length, caspase-3. After washing, slides were sequentially incubated with rat tissue specific horseradish peroxidase-polymer anti-rabbit antibody (Nichirei Corporation, Tokyo, Japan) for 30 min. The horseradish peroxidase was then reacted with DAB substrate for 3 min, and the sections were then counterstained with hematoxylin.

### Measurement of HO-1 activity

HO-1activity was measured in lung tissue by spectrophotometric determination of the rate of appearance of bilirubin as previously described [10]. HO-1 activity was reported as picomoles of bilirubin formed per milligram of protein per hour.

### Histologic Analysis

The lung lobe was stained with hematoxylin and eosin. The number of polymorphonuclear neutrophils in the lung interstitium was determined as the average number of polymorphonuclear neutrophils per high power field (×400). A minimum of 10 fields were randomly examined by an observer unaware of the protocol. Within each field, lung injury was scored according to (1) infiltration or aggregation of neutrophils in the airspace or vessel wall, and (2) thickness of the alveolar wall. Each assessment was graded 0, 1, 2, or 3, for no, mild, moderate, or severe injury, respectively. The resulting two scores were added and presented as the lung injury score [11].

### Experimental protocols

A total of 36 rats were randomized to receive an intraperitoneal vehicle (n = 18, dimethyl sulfoxide, Aldrich Sigma Chemical, St. Louis, MO, USA) or zinc protoporphyrin IX, a specific HO-1 activity inhibitor (n = 18, ZnPP, 20 mg/kg, Aldrich Sigma Chemical) injection 24 hours before this experiment. A pilot study showed that ZnPP at this dosage could suppress HO-1 activity.

The rat lungs were randomly assigned to the control group (n = 6), IR group (n = 6), or HCA+IR group (n = 6). The isolated lung preparation was allowed to equilibrate for 20 min. We recorded the baseline PAP, PVP and weight change, and measured the initial *K*_f_ for 7 min. Then we allowed all parameters to return to baseline values for 10 min.

In the IR group, as all parameters returned to baseline values, the lungs were subjected to 40 min of ischemia by stopping ventilation and perfusion and maintaining them in a deflated state. The lung preparations were kept at room temperature (25^o^C). The perfusion and ventilation were resumed and the measurement of K_f_ was repeated 90 min later. The lungs in the HCA+IR group were ventilated with a gas mixture of 10% CO_2_, 21% O_2_, and 69% N_2_ after the recirculating perfusion was established. The target pH and PCO_2_ values were around 7.1 and 70 mmHg, respectively,

### HO-1 siRNA for HCA experiment in A549 cells subjected to hypoxia-reoxygenation

Human alveolar epithelial cells (A549) (BCRC 60074, Food Industry Research and Development Institute, Hsinchu, Taiwan) were grown in 5% CO_2_ in F12K medium (Hyclone, Logan, UT, USA) containing 10% fetal bovine serum (Hyclone), 100 units/ml penicillin, and 100 µg/ml streptomycin. Cells were exposed to 24 h of hypoxia (1% O_2_-5% CO_2_-94% N_2_) followed by 4 h of 5% CO_2_-95% air (reoxygenation, H/R) or 10% CO_2_-90% air (HCA). The pH and PCO_2_ in the medium with HCA were 6.90 ± 0.01 and 72.7 ± 2.9 mm Hg, respectively. Transfection of cells with scrambled (negative control) or targeted siRNA was performed using Silencer® Select siRNA (Ambion, Invitrogen Life Technologies, Carlsbad, CA, USA) to achieve efficiencies of > 80%.

### Statistical analysis

The data are expressed as mean ± SD. Statistical differences between group means were determined with one-way or two-way repeated measures ANOVA, followed by a post hoc comparison using the Bonferroni post-test. Comparisons within each group for K_f_ were performed using paired Student’s *t*-tests. Significance was determined at the *P* < 0.05 level.

## Results

### PCO_2_ and pH in the perfusate

Baseline data obtained after 20 min of equilibration were compared with measurements made at the end of the experiment. The pH in the perfusate was significantly lower and the PCO_2_ was significantly higher at baseline in the IR+HCA and IR+HCA+ZnPP groups than in the IR and control groups (Table 1). At the end of the experiment, 10% CO_2_ was sufficient to maintain the target PCO_2_ and pH in the HCA groups.

**Table 1:** PCO_2_ and pH in the perfusate.

Variable	Control		Ischemia-reperfusion		Ischemia-reperfusion + hypercapnic acidosis	
	Vehicle	ZnPP	Vehicle	ZnPP	Vehicle	ZnPP
**pH**						
Baseline	7.39 ± 0.02	7.39 ± 0.01	7.40 ± 0.01	7.38 ± 0.02	7.20 ± 0.02^*+^	7.19 ± 0.01^*+^
Final	7.35 ± 0.02	7.38 ± 0.00	7.39 ± 0.01	7.37 ± 0.01	7.13 ± 0.03^*+^	7.16 ± 0.01^*+^
**PCO_2 (mm Hg)_**						
Baseline	35.6 ± 0.5	35.1 ± 0.4	35.3 ± 0.9	35.7 ± 0.8	67.4 ± 1.5^*+^	67.9 ± 4.5^*+^
Final	36.8 ± 0.8	36.0 ± 0.1	36.6 ± 0.5	36.5 ± 0.9	71.3 ± 0.6^*+^	71.8 ± 1.8^*+^

Data are expressed as mean ± SD. * significantly different from control (*P* < 0.05); + significantly different from ischemia-reperfusion (*P* < 0.05); using one-way ANOVA with Bonferroni post-test.

### Expression of HO-1 protein and activity in lung tissue

**Figure 1:**
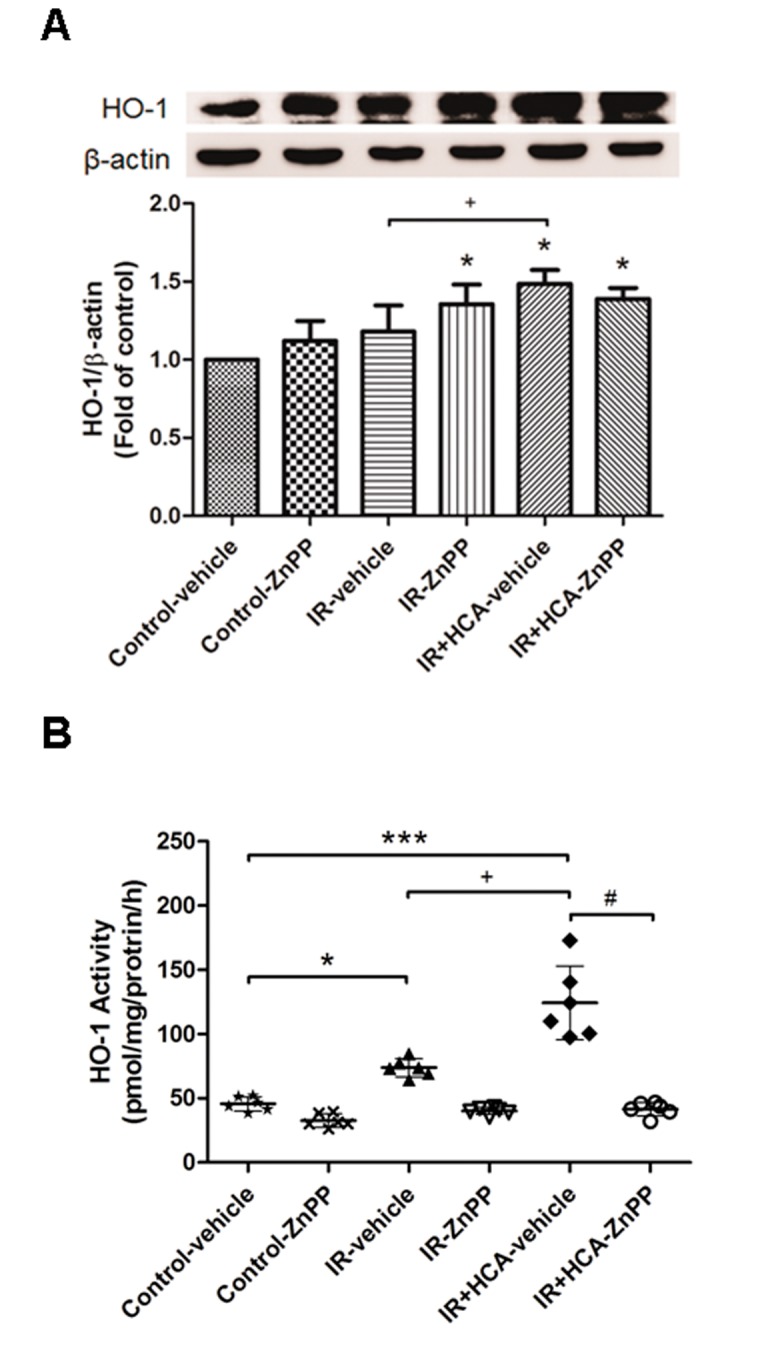
Effect of HCA and ZnPP on HO-1 protein expression and activity in lung tissue. Western blot and densitometry analysis were used for HO-1 protein expression (A). The induction of HO-1 protein by ischemia-reperfusion (IR) was accompanied by a marked increase in HO-1 activity (B). HCA further enhanced HO-1 protein expression and activity. ZnPP completely abolished HO-1 activity in either the absence or presence of HCA, although ZnPP also increased HO-1 protein expression. A representative blot is shown. Data are expressed as mean ± SD. *P < 0.05, ***P < 0.001, compared with control; ^+^ *P* < 0.05, compared with IR-vehicle; ^#^ *P* < 0.05, compared with IR+HCA, using one-way ANOVA with Bonferroni post-test.

First, we tested whether HCA alone induced HO-1 expression in isolated rat lungs. We found that HCA alone significantly induced HO-1 protein expression after 30 min perfusion without IR (Figure S1). IR significantly increased HO-1 protein expression compared with that in the control group (*P* < 0.05; Figure 1a). Furthermore, HCA treatment significantly enhanced lung HO-1 protein levels compared with the IR group. HO-1 protein expression in lung tissue of the control group and IR group tended to increase when ZnPP was added (Figure 1a). Further assessment of HO-1 activity revealed that the IR group had significantly increased HO-1 activity compared with the control group (*P* < 0.05; Figure 1b). The IR+HCA group had significantly increased HO-1 activity compared with the IR group. However, ZnPP treatment significantly reduced HO-1 activity in both the IR and IR+HCA groups (*P* < 0.05; Figure 1b).

### Pulmonary artery pressure

**Figure 2:**
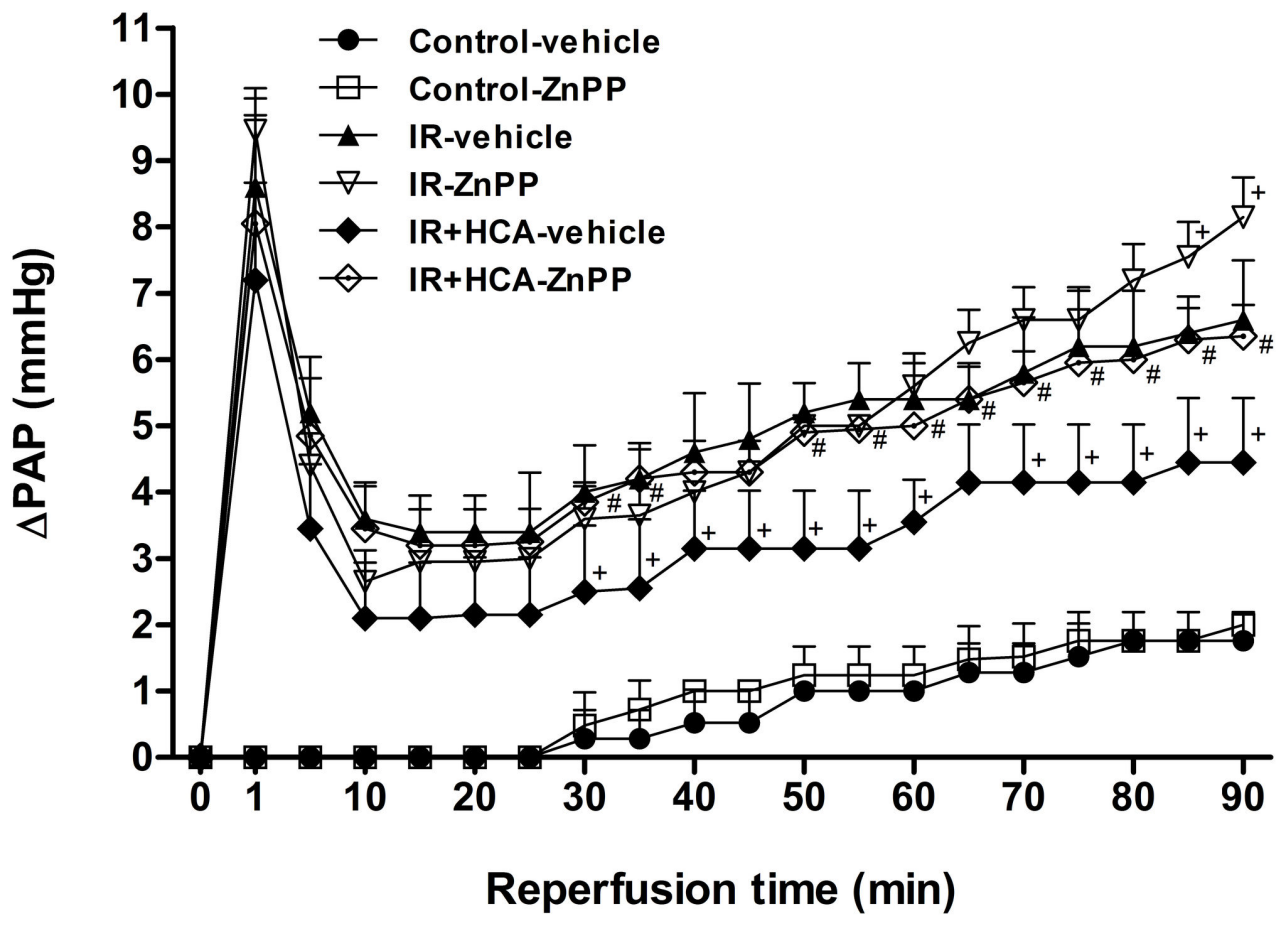
Effect of HCA and ZnPP on pulmonary artery pressure (ΔPAP). The PAP increased significantly in the ischemia-reperfusion (IR) group. The increase in the PAP was attenuated significantly by treatment with HCA, but not when ZnPP was added. Data are expressed as mean ± SD. ^+^significantly different from IR-vehicle (*P* < 0.05); *^#^* significantly different from IR+HCA (*P* < 0.05), using two-way ANOVA with Bonferroni post-test.

In the control group, the PAP showed almost no change during the 130 min observation period. In the IR group, the PAP increased after ischemia and then dropped to a trough at 20 min post reperfusion. At 90 min post reperfusion, the PAP was still significantly higher than in the control group and at baseline. Treatment with HCA significantly mitigated the increase in this later stage of PAP elevation. When ZnPP was added, the protective effect of HCA was significantly blocked (*P* < 0.05; Figure 2).

### Parameters of pulmonary edema

**Figure 3:**
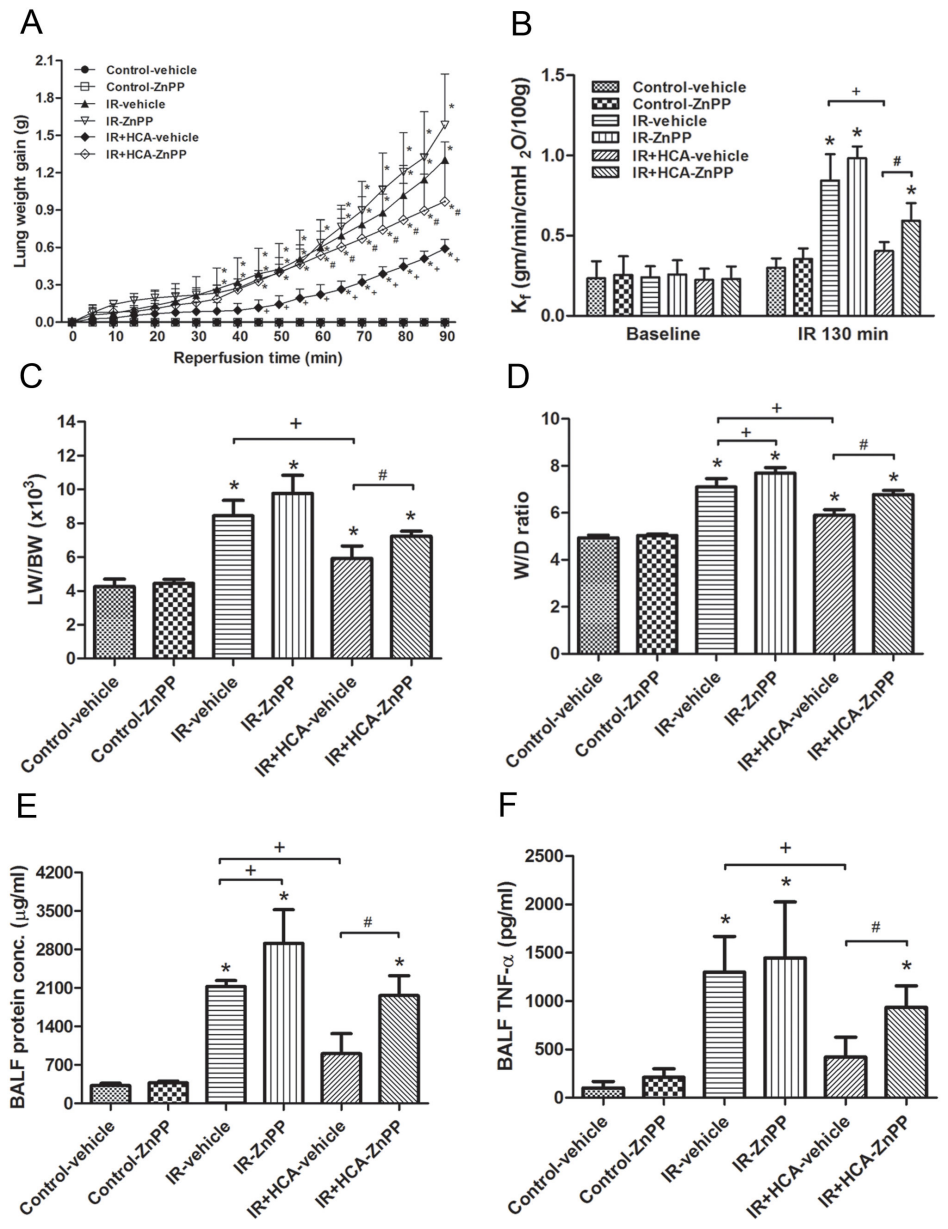
Effect of HCA and ZnPP on pulmonary edema and TNF-α level in bronchoalveolar lavage fluid. The lung weight gain (A), K_f_ (B), lung weight/body weight (LW/BW) (C) and wet/dry (W/D) weight ratios (D), protein concentration (E) and TNF-α level in the bronchoalveolar lavage fluid (F) increased significantly in the ischemia-reperfusion (IR) group. The increase in these parameters was significantly attenuated by treatment with HCA. The protective effect of HCA was partially abrogated by ZnPP treatment. Data are expressed as mean ± SD. *significantly different from control (*P* < 0.05); ^+^ significantly different from IR-vehicle (*P* < 0.05); *^#^* significantly different from IR+HCA (*P* < 0.05), using one-way or two-way ANOVA with Bonferroni post-test.

IR significantly increased lung weight gain (Figure 3a). This increase was attenuated by HCA treatment but the protective effect of HCA was partially blocked by the addition of ZnPP. Similarly, K_f_, LW/BW and W/D weight ratios, and the protein content in the BALF were significantly increased in the IR group (*P* < 0.05, Figure 3b–e), while HCA treatment resulted in a smaller increase in these parameters. However, addition of ZnPP partially blocked the protective effect of HCA.

### TNF-α production in BALF

The TNF-α levels in the BALF were significantly higher in the IR group compared with those in the control group (Figure 3f). TNF-α production in the BALF was inhibited by HCA in the IR group. However, the protective effects of HCA were partially abolished by treatment with ZnPP.

### Pathological findings

**Figure 4:**
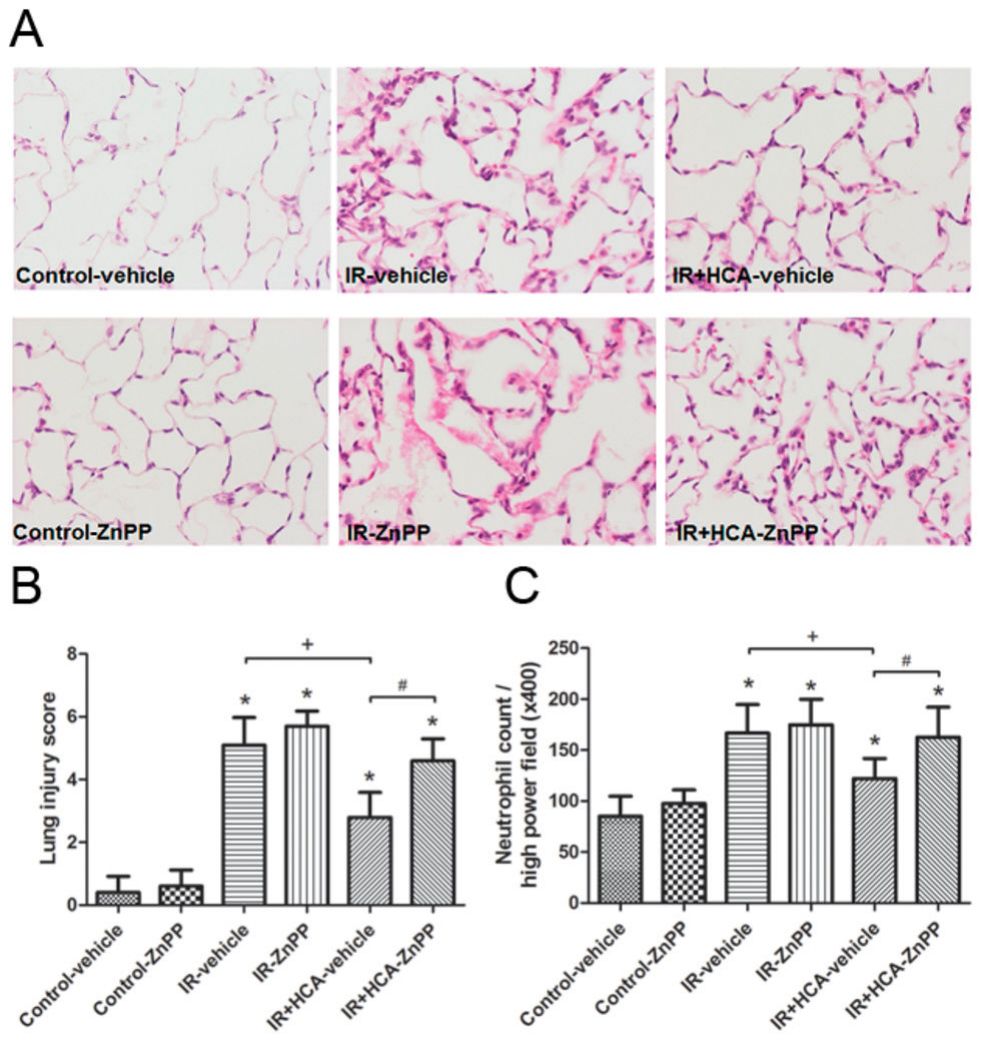
Effect of HCA and ZnPP on the histological appearance of lung tissue. As shown by a representative micrograph of lung tissue (400× magnification) (A), infiltrating neutrophils and septal widening were increased in the ischemia-reperfusion (IR) group. HCA treatment improved the histopathological changes, but the improvement was partially abolished by ZnPP treatment. The lung injury scores (B) and the numbers of neutrophils per high power field (400× magnification) (C) were significantly higher (*P* < 0.05) in the IR group than in the control group. HCA treatment significantly reduced the increase, but these improvements were partially abolished by ZnPP treatment. Data are expressed as mean ± SD. *significantly different from control (*P* < 0.05); +significantly different from IR-vehicle (*P* < 0.05); ^#^ significantly different from IR+HCA (*P* < 0.05), using one-way ANOVA with Bonferroni post-test.

Increased pulmonary vascular permeability and inflammatory responses were further confirmed by microscopic examination showing perivascular edema and increased inflammatory cell infiltration in the IR group compared with the control group (Figure 4a). The HCA-treated group showed decreased neutrophil infiltration (Figure 4c) and lung injury scores (Figure 4b). In contrast, the protective effect of HCA was partially abolished by pretreatment with ZnPP.

### Apoptosis

**Figure 5:**
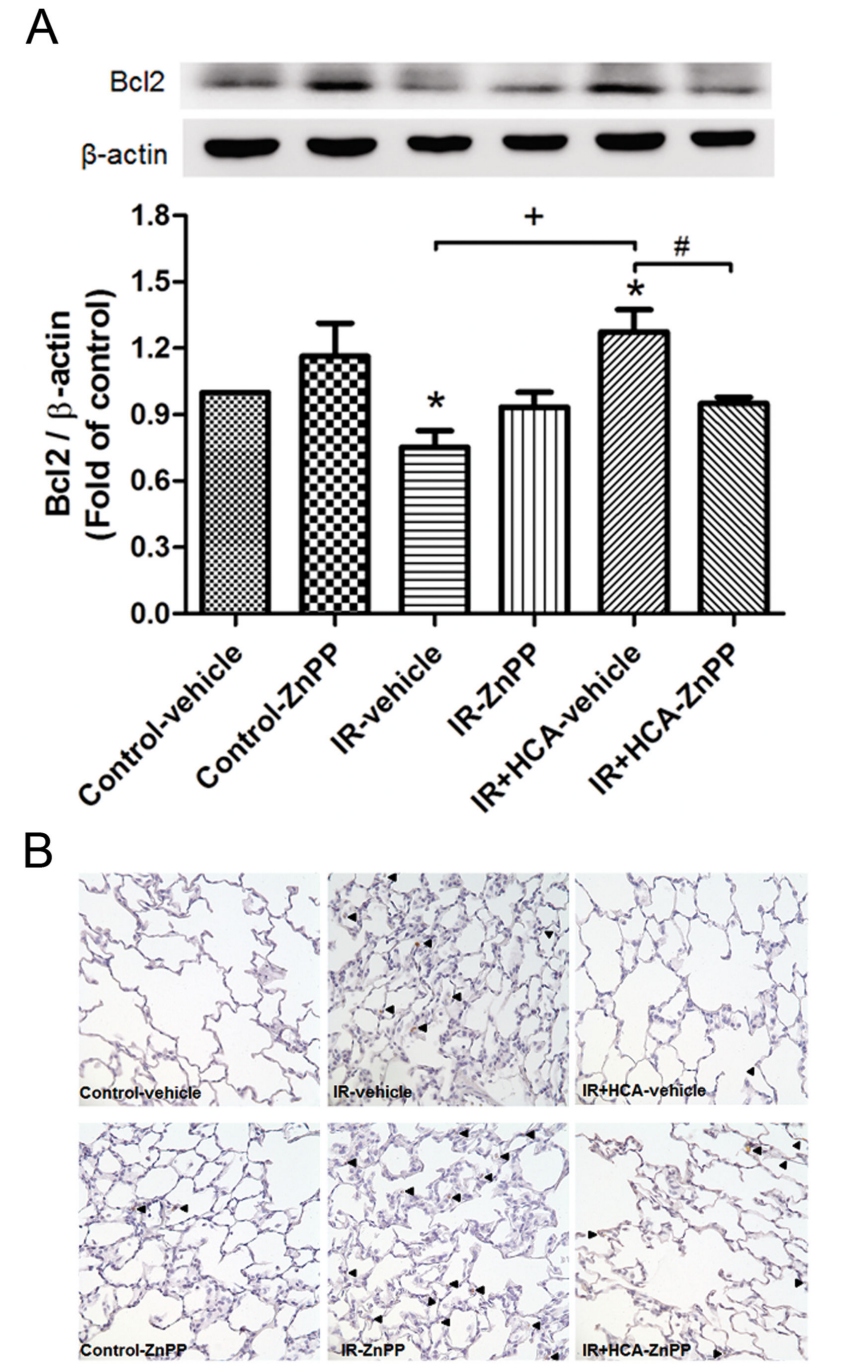
Effect of HCA and ZnPP on the expression of Bcl-2 and caspase-3 in the lung. (A) Western blot and densitometry analysis of Bcl-2 protein in lung tissue. β-actin served as a loading control for cytoplasmic proteins. A representative blot is shown. (B) Immunohistochemistry for active caspase-3 in the lung (200× magnification). Ischemia-reperfusion (IR) significantly decreased Bcl-2 protein expression and induced caspase-3 activation in the lung tissue. Hypercapnic acidosis (HCA) treatment significantly increased Bcl-2 protein expression and attenuated the signals for active caspase-3. When zinc protoporphyrin IX (ZnPP) was added, the protective effect was partially blocked. Data are expressed as mean ± D. *significantly different from control (*P* < 0.05); ^+^ significantly different from IR-vehicle (*P* < 0.05); ^#^ significantly different from IR+HCA (*P* < 0.05), using one-way ANOVA with Bonferroni post-test.

The Bcl-2 protein content in lung tissue was significantly lower in the IR groups than in the control groups, but was significantly increased upon HCA treatment. The protective effect was abolished by treatment with ZnPP (Figure 5a). The density of activated caspase-3-immunolabelled cells was significantly higher in the IR group compared with the control group. HCA treatment significantly decreased immunolabelled cells but the protective effect was partially reduced by the addition of ZnPP (Figure 5b).

### IKK-NFκB signaling pathway

**Figure 6:**
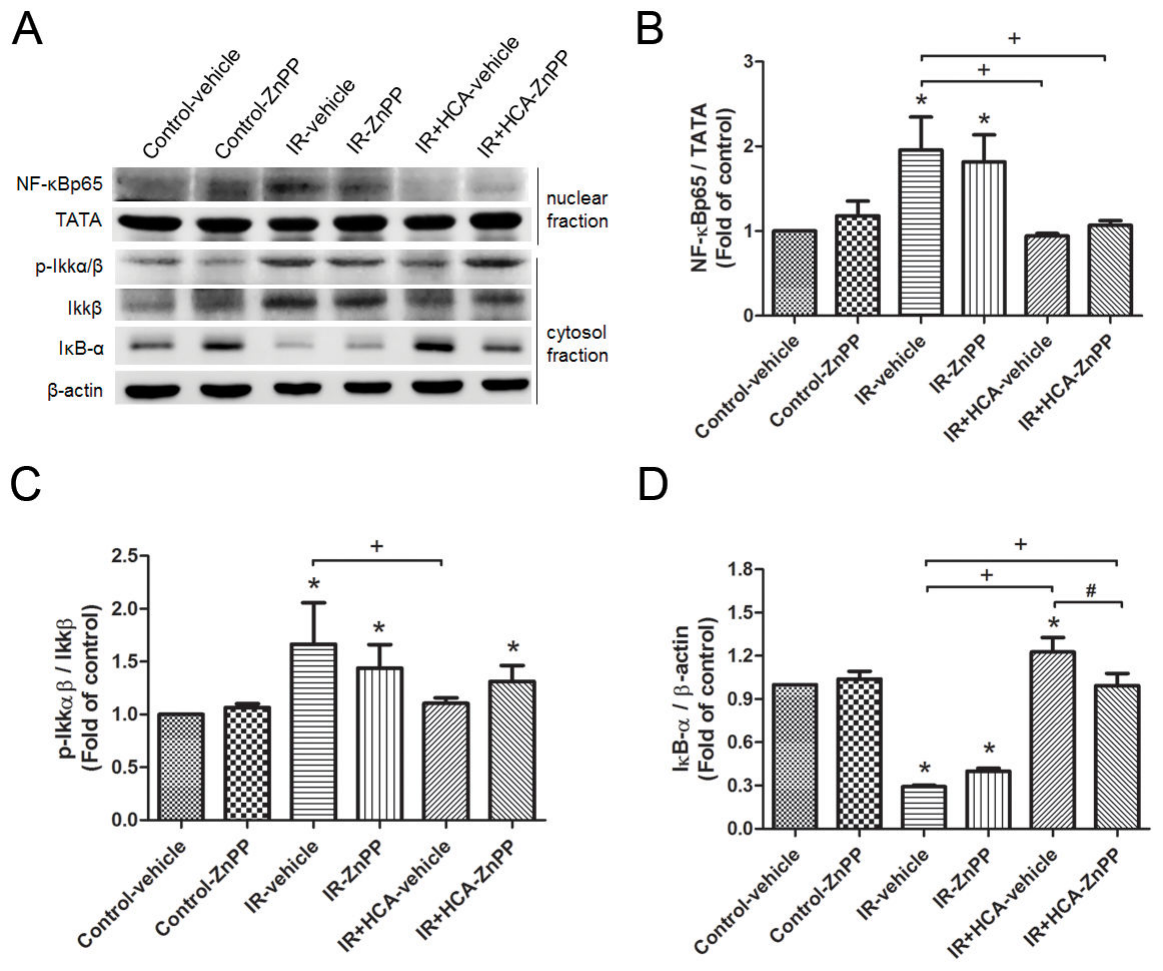
Effect of HCA and ZnPP for IKK-NFκB signaling pathway in lung tissue. Hypercapnic acidosis (HCA) increased the IκB-α level and reduced cytoplasmic phosphorylated IKK and nuclear NF-κB p65 levels in ischemia-reperfusion (IR)-induced lung injury. Zinc protoporphyrin IX (ZnPP) treatment partially attenuated the protective effect of HCA. TATA and β-actin served as loading controls for nuclear and cytoplasmic proteins, respectively. A representative blot is shown. Data are expressed as mean ± SD. *significantly different from control (*P* < 0.05); +significantly different from IR-vehicle (*P* < 0.05); ^#^ significantly different from IR+HCA (*P* < 0.05), using one-way ANOVA with Bonferroni post-test.

The cytoplasmic level of phosphorylated IKK and the nuclear level of NF-κB p65 were increased after IR injury (Figure 6), whereas the level of IκB-α was significantly suppressed in the IR group compared with the control group. HCA treatment restored the suppressed IκB-α level and reduced cytoplasmic phosphorylated IKK and nuclear NF-κB p65 levels. Treatment with ZnPP partially counteracted the protective effect of HCA.

### HO-1 siRNA in HCA-treated A549 cells subjected to H/R

**Figure 7:**
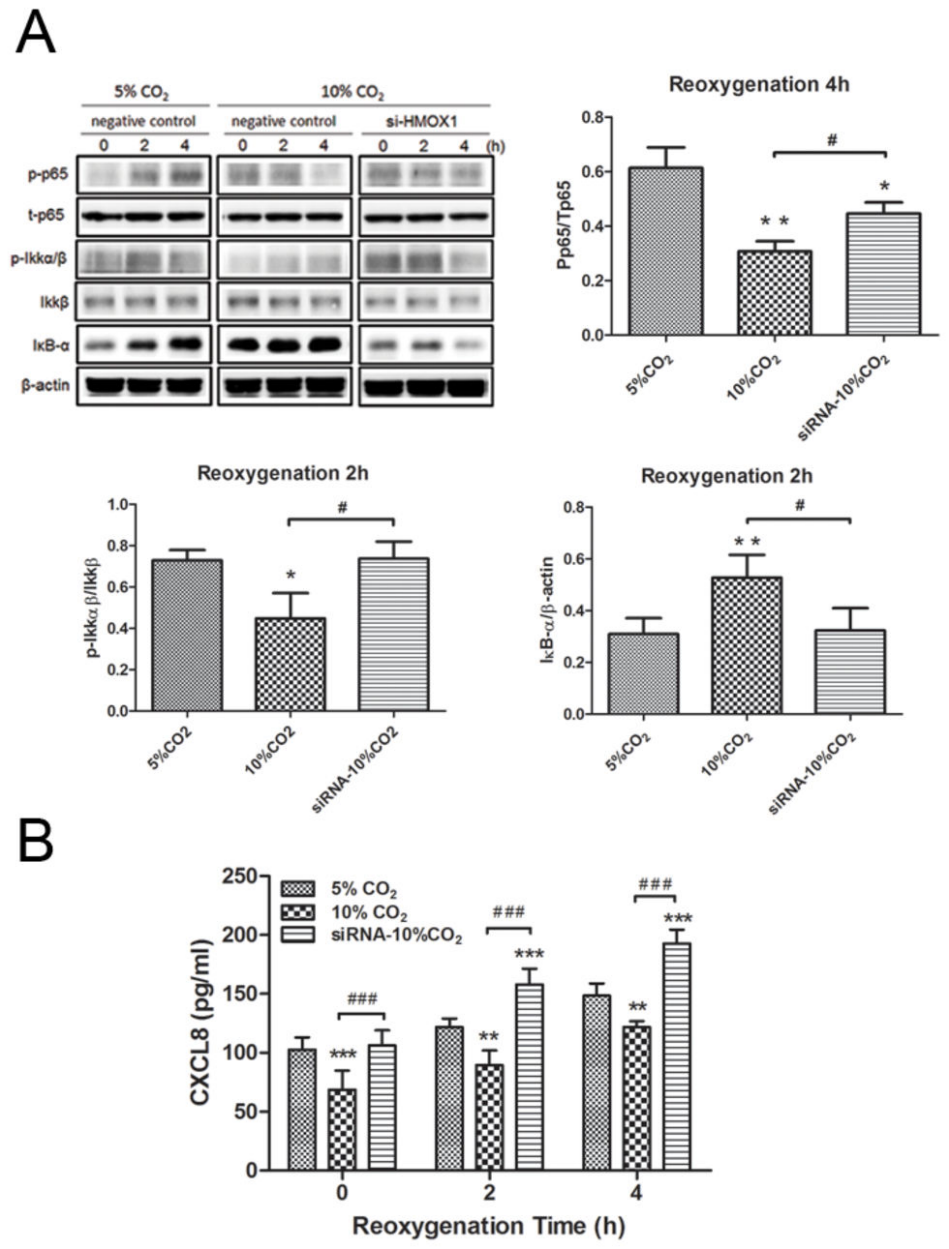
Effect of HCA and HO-1 siRNA in A549 cells subjected to hypoxia-reoxygenation (H/R). (A) HO-1 siRNA significantly attenuated the protective effect of HCA which reduced the increase of phosphorylated NF-κB p65 (p-p65) at 4 h, and p-IKK at 2 h, and the degradation of IκB-α at 2 h in A549 cells exposed to H/R. (B) HO-1 siRNA blocked the decrease of CXCL8 production by HCA in H/R-exposed A549 cells. A representative blot is shown. Data are expressed as mean ± SD. *P < 0.05, **P < 0.01, ***P < 0.001 versus 5% CO_2_; ^#^ *P* < 0.05, *^# # #^ P* < 0.001 versus 10% CO_2_, using one-way ANOVA with Bonferroni post-test.

HCA treatment in A549 cells without H/R for 30 min significantly increased HO-1 protein expression compared with 5% CO_2_. Transfection experiments revealed that HO-1 siRNA completely abolished HO-1 protein expression induced by 5% CO_2_ or HCA compared with the negative control (Figure S2). HCA treatment significantly reduced the H/R-induced increase of phospho-NF-κB p65 (p-p65) at 4 h, and p-IKK at 2 h in A549 cells (Figure 7a). At 2 h after H/R, the expression of IκB-α was also significantly increased by HCA. When the cells were transfected with HO-1 siRNA, the protective effect of HCA was significantly attenuated. In addition, HCA decreased H/R-induced epithelial production of the NF-κB-dependent CXCL8 (Figure 7b), but the effect was blocked by HO-1 siRNA. Furthermore, the Silencer select negative control siRNA did not affect H/R-induced NFκB signaling.

## Discussion

The major findings of the study are that (1) IR-induced lung injury significantly increased HO-1 protein expression and activity. HCA treatment significantly enhanced HO-1 protein expression and activity in IR-induced lung injury; (2) HCA attenuated IR-induced lung injury as evidenced by decreased pulmonary edema, reduced PAP, inhibition of the inflammatory response, attenuated apoptosis, improved lung pathology, and suppressed IKK-β phosphorylation, IκB-α degradation, and nuclear translocation of NF-κB, and (3) the protection provided by HCA can be damped by the HO-1 activity inhibitor, ZnPP. To further prove the effect of a selective pharmacological inhibitor, we took a genetic approach to confirm our hypothesis. Transfection of A549 cells with siRNA targeting the human HO-1 gene reversed the HCA-mediated inhibition of NF-κB signaling and inflammation. Taken together, our results suggest an important role for HO-1 in the lung protection provided by HCA.

In the present study HCA treatment preserved endothelial integrity and improved IR-induced pulmonary edema as demonstrated by decreased protein content in BALF, and a lower W/D lung ratio and LW/BW, as well as reduced K_f_. However, the reduced endothelial damage and vascular leakage caused by HCA were not completely abolished by the HO-1 inhibitor. Furthermore, apoptosis and IKK–NFκB signaling pathways were also partially blocked. We speculate that additional mechanisms are responsible for the protection afforded by HCA.

We identified an intriguing induction pathway for HO-1 in the protective mechanisms of HCA. HO-1 is the inducible form of heme oxygenase, the rate-limiting enzyme in heme degradation. Investigators are becoming increasingly aware that HO-1 has a very broad role in tissue injury and protection, including antioxidant, antiapoptotic, and anti-inflammatory actions. In our experiment, IR alone resulted in induction of HO-1 protein expression. However, IR still caused significant acute lung injury. This means that stress-induced HO-1 is not adequate to provide protection from tissue injury caused by IR. HCA further enhanced HO-1 expression and most importantly, attenuated lung injury induced by IR. In contrast, HO-1 inhibitor partially inhibited the protective effect of HCA. The present study corroborates previous investigations of IR-induced up-regulation of lung HO-1 expression, but this is not beneficial for lung injury [12,13]. Further induction of HO-1 protein and activity by ischemia postconditioning or administration of HO-1 adenovirus reduces lung reperfusion injury [12,13]. Our findings are also consistent with data reported from other experimental models and imply that only super-induction of HO-1 expression can exert significant beneficial effects in lung inflammation [14–16].

In our study, HO-1 protein expression was increased with ZnPP treatment (Figure 1a), whereas ZnPP inhibited HO-1 activity in the lung (Figure 1b). It is reported that HO-1 protein expression is increased by ZnPP, although it inhibits HO-1 enzyme activity [17,18]. One possible explanation for this phenomenon is that the *ho-1* gene is negatively controlled through product feedback inhibition. The suppression of HO-1 activity by ZnPP diminishes the availability of products of HO activity, thereby decreasing product feedback inhibition and, consequently, leading to an increase in *ho-1* gene activation. It should also be pointed out that induction of HO-1 protein expression by ZnPP would not inhibit NF-κB activation and attenuate lung injury because ZnPP effectively blocks HO-1 activity, and HO-1 exerts its protective effects through HO-1 activity.

IR lung injury has been demonstrated to be associated with the expression of TNF-α and neutrophil infiltration in the lung [11,19]. Induction of HO-1 protein expression is reported to downregulate lipopolysaccharide-stimulated macrophages from release of proinflammatory mediators such as TNF-α [20]. In addition, HO-1 is capable of suppressing neutrophil rolling, adhesion and migration, thereby preventing neutrophil recruitment to the site of inflammation [21]. In the present study, our findings were comparable with these observations that HO-1 overexpression may contribute to the beneficial effects of HCA by reducing the production of TNF-α and neutrophil infiltration.

NF-κB is an important transcription factor required for regulation of the gene expression of cytokines, chemokines, and adhesion molecules [22]. Investigators have reported the negative effect of HO-1 on NF-κB activation [23,24]. Our previous studies and this study showed that IR injury induced NF-κB activation and translocation [11,19]. Treatment with ZnPP, a selective inhibitor of HO-1 activity in this study, partially inhibited the decrease of nuclear NF-κB levels that was caused by HCA treatment, confirming the involvement of this enzyme in NF-κB signaling suppression by HCA.

Apoptosis is the process of programmed cell death and has been implicated in the pathogenesis of pulmonary IR injury [25]. The inhibition of apoptosis and related cell death pathways could reduce IR lung injury [25]. HCA has been shown to decrease inflammatory responses and apoptosis in rabbits with IR lung injury [26]. Although the exact mechanism by which HCA attenuates apoptosis is unknown, it is speculated that HCA leads to diminished apoptosis-specific DNA fragmentation [26]. In this study, HCA seemed to attenuate apoptosis via HO-1 induction. Many studies have revealed that upregulation of HO-1 can inhibit apoptosis in pulmonary and vascular cells mediated by TNF-α and in preclinical models of lung injury [27], which is consistent with our result that decreased caspase-3 expression in HCA-treated animals was blocked by ZnPP treatment. The cellular mechanisms by which HO-1 exerts cytoprotective functions in pulmonary IR injury could involve expression of antiapoptotic proteins [27]. HO-1 has been shown to induce the expression of Bcl-2 protein and provide protection in a cardiac cold IR model [28]. Indeed, ZnPP treatment inhibited the expression of Bcl-2 protein that was significantly increased by HCA in this study. Nonetheless, further investigation is needed to determine whether other antiapoptotic proteins play important roles in lung protection after HCA treatment.

Biliverdin and CO, end-products of HO-1 catalysis, possess immunomodulatory, antiapoptotic and anti-inflammatory properties; they inhibit NF-κB activation and attenuate various modes of lung injury [6,29]. HO-1 induction is also coupled to increased availability of ferritin, leading to rapid conjugation and removal of free iron, which is another source of potential oxidative stress [6]. It is likely that the protective properties of HO-1 afforded by HCA are partially mediated by products of HO-1 metabolism rather than by HO-1 itself. Further studies should clarify their individual roles in the protection afforded by HCA.

In summary, we demonstrated that HCA reduced lung IR injury in isolated rat lungs by decreasing infiltration of neutrophils, lung edema, TNF-α production in the BALF, IKKβ phosphorylation and nuclear translocation of NFκB, and increasing HO-1 protein expression. The protective effect of HCA was dampened by the presence of ZnPP, an HO-1 activity inhibitor. In addition, HO-1 siRNA significantly reversed HCA-mediated inhibition of NF-κB signaling in A549 cells subjected to H/R. These data support the concept that HCA-induced HO-1 expression is at least partly responsible for the anti-inflammatory effects of HCA. There remain a multitude of candidate pathways to account for the therapeutic efficacy of HCA. Further studies should provide a better understanding of this complex protective mechanism of HCA.

## Supporting Information

Figure S1 **The effect of hypercapnic acidosis (HCA) in HO-1 protein expression in the lung without ischemia-reperfusion (IR).** Western blot analysis revealed that HCA treatment significantly increased HO-1 protein expression in isolated rat lungs without IR for 30 min when compared with 5% CO_2_. A representative blot is shown. **P < 0.01, ***P < 0.001, using one-way ANOVA with Bonferroni post-test.
(TIFF)

Figure S2 **Western blot and densitometry analysis for HO-1 protein expression in A549 cell.** Hypercapnic acidosis (HCA) treatment in A549 cell without hypoxia-reoxygenation for 30 min significantly increased HO-1 protein expression when compared with 5% CO_2_. HO-1 siRNA significantly abolished HO-1 protein expression induced by 5% CO_2_ or HCA compared to negative control. A representative blot is shown. Data are expressed as mean ± SD. ***P < 0.001, using one-way ANOVA with Bonferroni post-test.
(TIFF)
