# Supplementary figures and images for: Protective Effect of Hypercapnic Acidosis in Ischemia-Reperfusion Lung Injury Is Attributable to Upregulation of Heme Oxygenase-1
Source: PLoS One. 2013 Sep 10;8(9):e74742. doi: 10.1371/journal.pone.0074742 (PMC3769390; doi:10.1371/journal.pone.0074742)

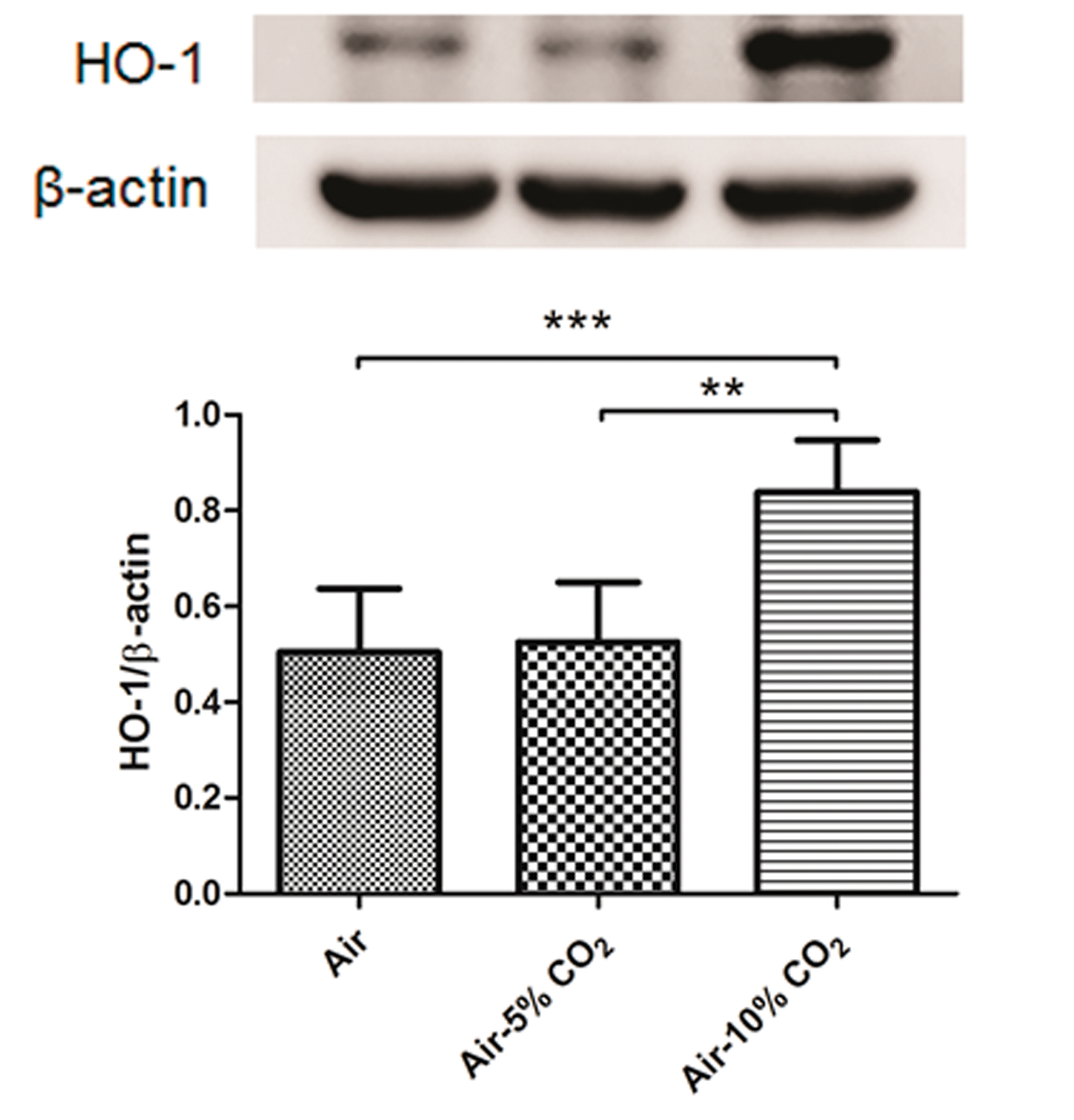

Supplement: Figure S1 — The effect of hypercapnic acidosis (HCA) in HO-1 protein expression in the lung without ischemia-reperfusion (IR). Western blot analysis revealed that HCA treatment significantly increased HO-1 protein expression in isolated rat lungs without IR for 30 min when compared with 5% CO2. A representative blot is shown. **P < 0.01, ***P < 0.001, using one-way ANOVA with Bonferroni post-test. (TIFF) [file pone.0074742.s001.tiff]

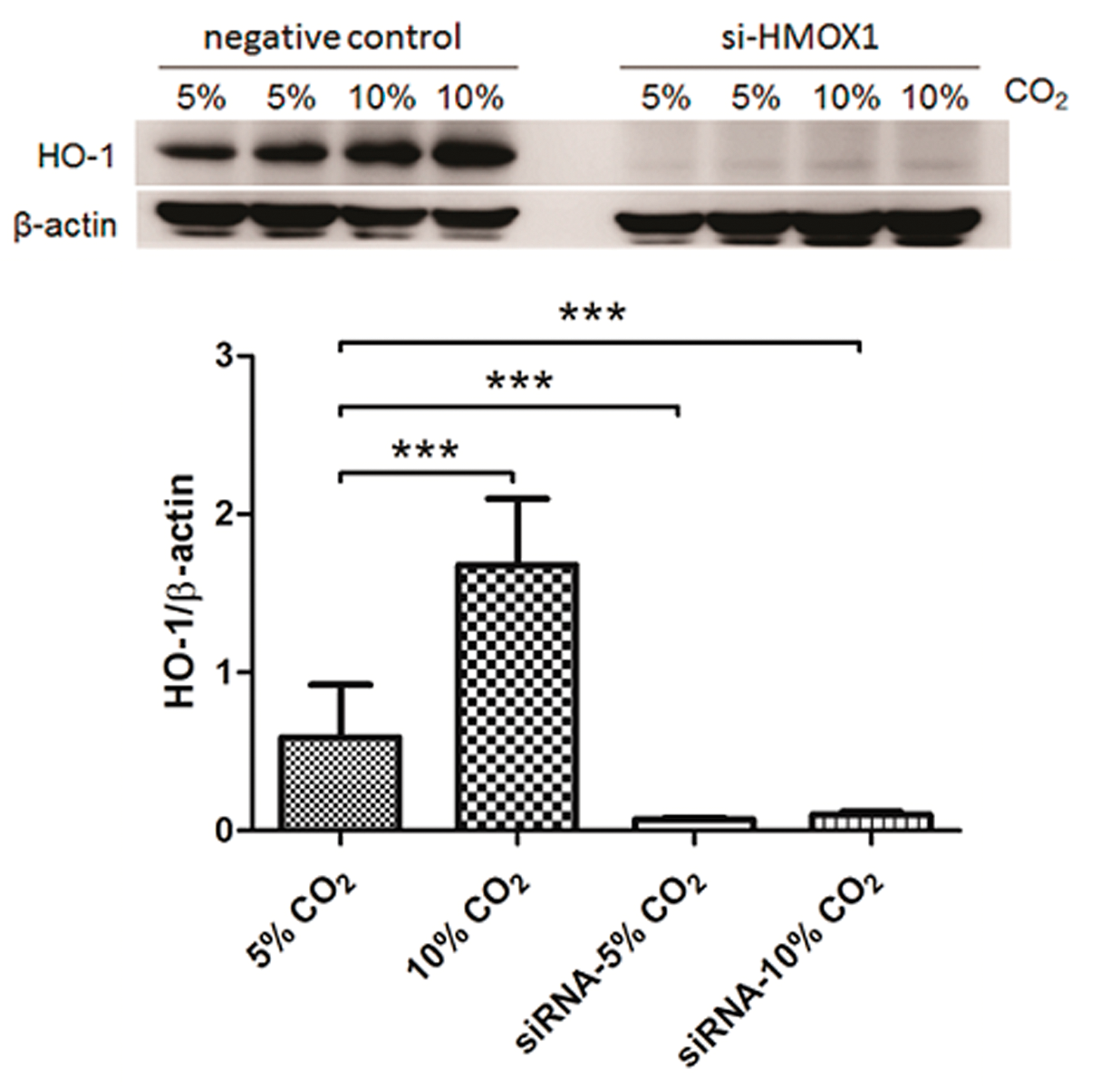

Supplement: Figure S2 — Western blot and densitometry analysis for HO-1 protein expression in A549 cell. Hypercapnic acidosis (HCA) treatment in A549 cell without hypoxia-reoxygenation for 30 min significantly increased HO-1 protein expression when compared with 5% CO2. HO-1 siRNA significantly abolished HO-1 protein expression induced by 5% CO2 or HCA compared to negative control. A representative blot is shown. Data are expressed as mean ± SD. ***P < 0.001, using one-way ANOVA with Bonferroni post-test. (TIFF) [file pone.0074742.s002.tiff]
